# Novel approaches to energize microbial biocatalysts

**DOI:** 10.1111/1462-2920.16254

**Published:** 2022-11-15

**Authors:** Gonzalo Durante‐Rodríguez, Manuel Carmona, Eduardo Díaz

**Affiliations:** ^1^ Department of Microbial and Plant Biotechnology Centro de Investigaciones Biológicas Margarita Salas‐CSIC Madrid Spain

## Abstract

An efficient and cheap energization of microbial biocatalysts is essential in current biotechnological processes. A promising alternative to the use of common organic or inorganic electron donors is the semiconductor nanoparticles (SNs) that absorb light and transfer electrons (photoelectrons) behaving as artificial photosynthetic systems (biohybrid systems). Excited photoelectrons generated by illuminated SNs are highly reductive and readily accepted by membrane‐bound proteins and electron shuttles to drive specific cell reduction processes and energy generation in microbes. However, the operational mechanisms of these hybrid systems are still poorly understood, especially at the material–microbe interface, and therefore the design and production of efficient biohybrids are challenging. Some major limitations/challenges and future prospects of SNs as microbial energization systems are discussed.

## INTRODUCTION

Hungarian physiologist Albert Szent‐Györgyi, who won the Nobel Prize in 1937 for the discovery of vitamin C, stated that “life is nothing but an electron looking for a place to rest.” This sentence summarizes very accurately that a flux of electrons is required for life maintenance. This flux initiates with the entrance of electrons in the organism from one electron donor. Microbes have evolved to accept electrons from a huge amount of donors. Organic compounds or inorganic materials, such as metals, metalloids, sulphur or hydrogen, transfer their electrons to carriers which initiate the electron flux that finally provides the energy needed by the cells. In current biotechnological processes, an efficient and cheap energization of the biocatalysts is a key issue for achieving competitive yields. In this sense, a promising alternative to the use of common organic or inorganic electron donors are the semiconductor nanoparticles (SNs) that absorb light and transfer electrons (photoelectrons) behaving as artificial photosynthetic systems that self‐repair and self‐generate (Kumar et al., 2019). Living‐cells, for example, bacteria, yeast, can be coupled with SNs generating the so‐called biohybrid systems (Cheng et al., 2021; Kornienko et al., 2016).

**FIGURE 1:**
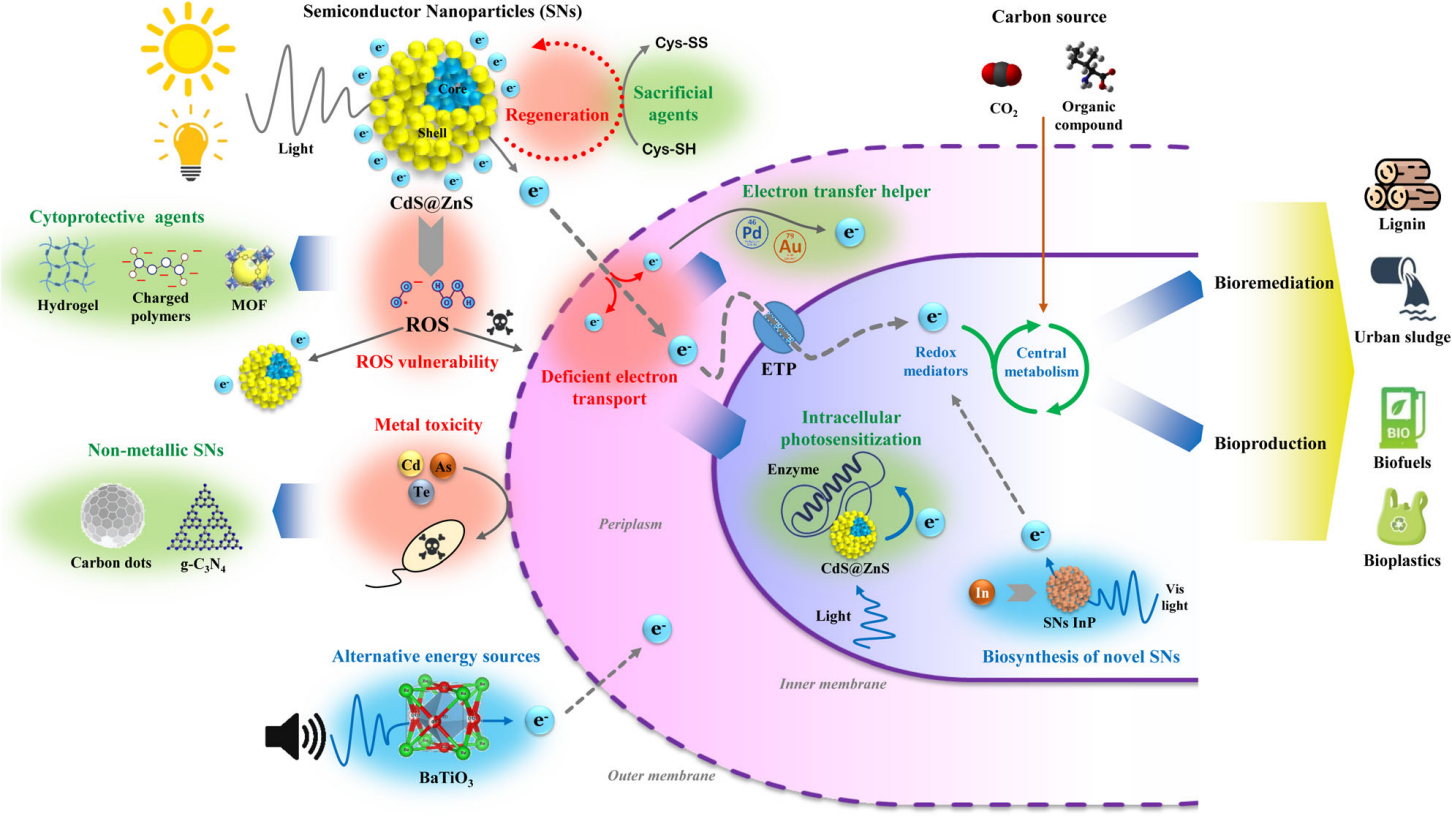
Main challenges on the use of semiconductor nanoparticles (SNs) as energization systems of microbial biocatalysts. The main limitations/challenges are indicated in red. The main current strategies to overcome these limitations are indicated in green. Future prospects are shadowed in blue. Au, gold; CdS@ZnS, core@shell nanoparticle; ETP, electron transfer proteins; g‐C_3_N_4_, graphitic carbon nitrides; InP, indium phosphide nanoparticles; MOF, metal organic framework; Pd, palladium; ROS, reactive oxygen species.

Inorganic SNs are promising light harvesters due to its solar energy conversion efficiency of about 20% (with respect to a theoretical limit of 33.7%), high surface area, ease of interaction at microbe–material interface because of their nanoscale dimensions (1–20 nm), and biocompatibility with living cells (Gallardo‐Benavente et al., 2019; Mal et al., 2016; Órdenes‐Aenishanslins et al., 2019; Sahoo et al., 2020). Among the different SNs produced, those constituted by calcogenic elements, such as CdS, ZnS, CdSe and CdTe, have attracted more attention, especially CdS since they possess a relatively narrow band gap (2.4 eV) and enhanced performance for visible light absorption (Dong et al., 2020; Mal et al., 2016). The elements of SNs can be arranged in only one layer (core) or in a more complex structure that involves a shell that encloses the core, maintaining its chemical stability and improving the surface activity (Órdenes‐Aenishanslins et al., 2019) (Figure 1).

## BIOPRODUCTION OF SNs

SNs can be synthesized by physical, chemical or biological means. The physical synthesis methods are generally low‐cost and eco‐friendly, but the resulting SNs usually harbour high surface defects, low stability, and poor quantum yield (Zeng et al., 2012). Chemical synthesis methods are the traditional approaches to prepare SNs monodispersed and with good optical performance, but these strategies employ harsh reaction conditions with the use of pollutant solvents, and surface modification is required for the achievement of desirable functions (Zhou et al., 2015). Conversely, bioproduction of SNs is an eco‐friendly process that requires low energy costs and the SNs produced have high biostability and biocompatibility. The first report describing the bioproduction of SNs was that dealing with CdS synthesis by the yeasts *Candida glabrata* and *Saccharomyces pombe* (Dameron et al., 1989). Since then, a good number of publications reported a wide variety of microorganisms, including some sulphate reducing bacteria, genetically modified *Escherichia coli*, *Rhodopseudomonas palustris*, *Acidithiobacillus* sp. or *Pseudomonas* spp. (Choi & Lee, 2020). Biosynthesis of SNs is a complex process since it requires the close interaction of the elements that compose the SNs in a specific oxidation state (Órdenes‐Aenishanslins et al., 2020). For example, for the bioproduction of CdS it is necessary that bacteria generate sulphide anions (S^2−^) that react with cadmium cations (Cd^2+^). Sulphide anions are present in the thiol groups of molecules rich in cysteine that, in addition to serving as sulphur source, stabilize the SNs (Órdenes‐Aenishanslins et al., 2020). It is assumed that the mechanism responsible for the formation of CdS in bacteria is based on the metabolic production of hydrogen sulphide (H_2_S) by desulfhydrase enzymes which break the carbon–sulphur bonds of cysteine (Ulloa et al., 2016). Therefore, the use of bacteria as biofactories for the production of SNs (e.g., CdS or ZnS) relies on the exogenous addition of l‐cysteine to the culture medium (Farzin & Abdoos, 2021; Órdenes‐Aenishanslins et al., 2020).

## SNs AS CELL ENERGIZERS

Excited photoelectrons generated by illuminated SNs are highly reductive and readily accepted by membrane‐bound proteins (e.g., cytochromes, ferredoxins, hydrogenases, etc.) and electron shuttles (e.g., quinones) to drive specific cell reduction processes and energy generation in microbes (Chen et al., 2022; Kornienko et al., 2016) (Figure 1). Hybrid living systems that harvest light energy through SNs can energetically enhance the conversion of inorganic materials, for example, CO_2_, to high value‐added organic chemicals, polymers, fertilizers and energy products (e.g., H_2_), while they are self‐regenerating, with low waste generation, and operating at an efficiency (product yield based on solar energy input) higher than 80% (Fang et al., 2020; Gupta et al., 2021; Tremblay et al., 2020). For instance, incorporating biocompatible CdS SNs to *R. palustris* cells leads to an increase of the intracellular production of ammonia and l‐amino acids from extracellular inorganic nitrogen gas when photoelectrons are generated under light (Wang et al., 2019). Different biohybrid outcomes can be achieved depending on the host strains and target products. Thus, whereas a living hybrid composite consisting on CdS/ZnS SNs and *Azotobacter vinelandii* promoted conversion of N_2_ to NH_3_ under illumination with green wavelengths, assembly of InP/ZnS SNs with *Cupriavidus necator* allowed production of bioplastic (polyhydroxybutyrate, PHB) by incorporating CO_2_ under illumination with red wavelengths (Ding et al., 2019). CdS SNs serve also as electron donors for denitrifying proteins in *Thiobacillus denitrificans* to enhance gaseous nitrous oxide production from nitrate under light‐driven conditions (Chen et al., 2019). These studies reveal that SNs represent an interesting strategy to confer to non‐photosynthetic bacteria the ability of light utilization (Cestellos‐Blanco et al., 2021).

The exogenous addition of chemically synthesized SNs has been employed to increase also the yield in heterotrophic bioprocesses including polyhydroxybutyrate (PHB) synthesis from fructose by *Cupriavidus necator* and shikimate (a precursor for drugs and many fine chemicals) production from glucose by *Saccharomyces cerevisiae* (Chen et al., 2020; Guo et al., 2018; Xu et al., 2021). Hydrogenotrophic bacteria, for example, *C. necator*, can also benefit of the photocatalytic capacity of CdS SNs for production of hydrogen by splitting water under visible light irradiation (Cheng et al., 2018). SNs have been also used to enhance bioremediation of refractory organic pollutants. For instance, it was reported azo dye degradation by CdS–*Geobacter sulfurreducens* biohybrids under intermittent light illumination through a synergistic effect between a key *c*‐type outer membrane cytochrome that facilitates photoelectron transfer and the adsorption/photocatalysis (mediated by the formation of reactive oxygen species) of large organic pollutants into low‐molecular‐weight by‐products (Huang et al., 2019).

A drawback of the metal‐based SNs is that they leach toxic metals after photocorrosion which may pose environmental hazards when applied at industrial scale (Ding et al., 2019; Huang et al., 2019). Additionally, the wide range of band gaps comprising metal‐based SNs often rely on high‐intensity light sources (e.g., UV‐irradiation) for effective excitation, which limits their application in living biohybrids (Fang et al., 2020). As an alternative, metal‐free carbon‐based light harvesters, such as graphitic carbon nitrides (g‐C_3_N_4_) and carbon dots (CDs), that exhibit low toxicity and cost, high water solubility, photostability and tunability (with an absorption wavelength around 600 nm), have been developed (Liu et al., 2020; Figure 1). g‐C_3_N_4_ harvested solar energy and supplied reducing equivalents to *C. necator* for efficient bioplastic (PHB) production. Coupling g‐C_3_N_4_ with catalase, efficient for degradation of H_2_O_2_ generated during photocatalytic water splitting, improved further the production of PHB (Tremblay et al., 2020). On the other hand, the CDs size/geometry match the active sites of some enzymes generating a stronger light‐harvesting centre in certain biohybrid systems, for example, in hydrogenases for scalable photocatalytic H_2_ production (Holá et al., 2020).

## LIMITATIONS OF THE SNs AND FUTURE PROSPECTS

SNs represent a powerful tool for energization of microbial catalysts. Unlike other processes like electrosynthesis, biohybrids are stand‐alone systems that do not require additional electron inputs. However, the working mechanism of these hybrid systems is still poorly understood, especially at the material–microbe interface, and the design and production of efficient biohybrids is challenging. Here we list some major limitations/challenges and future prospects of SNs as microbial energization systems (Figure 1).

When using extracellular SNs–bacterial interfaces only a small fraction of the released electron flow from the excited SNs outside of the cell is effectively transferred (after crossing the outer membrane and periplasm) to the electron transport molecules located in the cell (inner) membrane (Kumar et al., 2017; Liu et al., 2018). Some approaches to try to solve this problem are, for instance, the use of engineered conductive nanomaterials such as graphene or carbon nanotubes between cell components and excited SNs (Yin & Wu, 2019). Another strategy is to target the SNs to specific cell locations in the envelope to ensure the interfacial electron transfer. For instance, it has been reported that the localization of palladium (Pd) or gold (Au) nanoparticles into the periplasm helps to fill voids between cytochromes (Wu et al., 2011). Intracellular photosensitization is another recent approach based on the use of SNs that are passively inserted into a living cell. These harvesters, for example, nanoparticles with a zinc sulphide shell that enhances binding to certain proteins, attach themselves to desired enzymes (native or specifically tagged to facilitate binding to SNs) and match their electrochemical potential so that they can be triggered by a specific wavelength of light (Ding et al., 2019; Figure 1).

Although the positive effect of adding exogenous SNs on bacterial growth/production has been shown, the effects of photo‐excitation and electron transference from endogenous SNs to cellular elements during bacterial growth have been poorly studied so far. Thus, it would be worthwhile to explore the possibility to synthesize SNs in bacteria as an alternative/complementary way of cell energization. Synthetic biology approaches to boost electron transfer, for example, modifying conductive cytochromes, intracellular redox mediators, NADH pool, SNs–enzyme nanobiohybrids and so on, will facilitate the tailoring of living hybrid systems to target specific applications (Chen et al., 2022; Figure 1).

Living hybrid composites are vulnerable and sensitive to the external environment, for example, reactive oxygen species produced during photocatalysis or membrane destruction, hence, it is extremely important to protect their integrity. Sacrificial agents (such as cysteine) are often added to bacteria–SNs systems to quench holes generated by the absorption of light (Sakimoto et al., 2016). New materials are developing as efficient and recyclable agents for protecting living biohybrids from reactive oxygen species while improving their mechanical strength, adaptability and thermal stability. Current and future cytoprotective strategies rely on the wrapping of microbes with charged polymers, hydrophilic hydrogels (e.g., alginate), metal–organic frameworks (MOFs), porous organic polymers (POPs) and nanozymes (e.g., superoxide dismutase, glutathione peroxidase or catalase, as effective reactive oxygen species‐scavengers) (Chen et al., 2018; Cheng et al., 2021; Jo et al., 2020; Figure 1).

The photocatalytic performance of SNs can be improved significantly by optimizing their morphology, specific surface area and charge, and crystallinity. For instance, spherical nanoparticles are more efficacious relative to other morphologies given the low surface area‐to‐volume ratio of a sphere, which contributes to maximize their biocompatibility and interactions with the cell membrane and, hence, foster electron transference to enzymatic mediators and metabolic charge carriers (Deng et al., 2020; Sakimoto et al., 2016). Although these features are difficult to optimize through the standard chemical synthesis of SNs, they could be approached through their biological production (Cheng et al., 2018; Jiang et al., 2020). It would be interesting also to biosynthesize other types of SNs endowed with new optimal properties as photocatalysts. Indium phosphide (InP) nanoparticles have been recently reported to absorb visible light, which makes them more efficient than other SNs that can only absorb short wave‐length (UV) light which comprises at most 5% of the incident sunlight and can cause DNA damage and cell death (Guo et al., 2018).

To gain further insights into the genetic information and fundamental processes that underlie the bio‐hybrid coupling to create more seamless interfaces omic approaches are required. A transcriptomic analysis of *Clostridium autoethanogenum* growing in the presence of chemically synthesized CdS added to the growth medium has been performed, and it revealed the up‐regulation of the Rnf complex which couples ferredoxin oxidation with NAD^+^ reduction (Jin et al., 2021). Proteomics and metabolomics performed in the non‐photosynthetic *Moorella thermoacetica*–CdS inorganic hybrid system were also performed showing up‐regulation of several proteins (e.g., flavoproteins, ferredoxins, NADP dehydrogenase) involved in the electron transfer from the photo‐excited CdS nanoparticles. It is thought that electrons taken up by these enzymes are further shuttled across the cell membrane by quinones (Jin et al., 2021; Zhang et al., 2020). However, it becomes essential to expand omics approaches to other type of microorganisms to understand the global impact of SNs on microbial physiology, as well as to know for the first time the changes that occur in cells producing SNs. The knowledge on the molecular determinants responsible for the biosynthesis of SNs will be then used for the rational design of novel SNs (Santos‐Merino et al., 2019), paying special attention to those more adaptive to environmental conditions and more versatile for electron transfer (Jensen et al., 2010; Yang et al., 2015).

Finally, alternative mechanisms of excitation of SNs need to be explored. Some metallic nanoparticles such as BaTiO_3_ (barium titanate) are piezoelectric and, hence, sound at determined frequency promotes electron transfer (Marino et al., 2018). This phenomenon can be explored in microbes paving the way for an unprecedented generation of sonic‐sensitized biocatalysts (Figure 1).

In summary, SNs–biological hybrid systems represent sustainable, efficient and versatile chemical synthesis platforms by integrating the light‐harvesting properties of semiconductors with the synthetic potential of biological cells (Guo et al., 2018). Owing to the synergist benefits of interdisciplinary research (microbiology, molecular biology, electrochemistry, materials science, environmental science), the design of future living hybrid systems overcoming the major drawbacks of low efficiency, tunability, scalability, tolerance to environmental stressors and economic feasibility, has the potential to impact several commercial processes while addressing global sustainability issues.

## AUTHOR CONTRIBUTIONS

**Gonzalo Durante‐Rodriguez:** Conceptualization (equal); writing – original draft (equal); writing – review and editing (equal). **Manuel Carmona:** Conceptualization (equal); writing – original draft (equal); writing – review and editing (equal). **Eduardo Diaz:** Conceptualization (equal); funding acquisition (lead); writing – original draft (equal); writing – review and editing (equal).

## CONFLICT OF INTEREST

The author declares no potential conflict of interest.

## ETHICS STATEMENT

The content and authorship of the submitted manuscript has been approved by all authors, and all prevailing local, national and international regulations and conventions, and normal scientific ethical practices, have been respected.
